# High throughput mathematical modeling and multi-objective evolutionary algorithms for plant tissue culture media formulation: Case study of pear rootstocks

**DOI:** 10.1371/journal.pone.0243940

**Published:** 2020-12-18

**Authors:** Saeid Jamshidi, Abbas Yadollahi, Mohammad Mehdi Arab, Mohammad Soltani, Maliheh Eftekhari, Jalal Shiri

**Affiliations:** 1 Department of Horticultural Science, Faculty of Agriculture, Tarbiat Modares University (TMU), Tehran, Iran; 2 Department of Horticultural Sciences, College of Aburaihan, University of Tehran (UT), Tehran, Iran; 3 Department of Irrigation and Drainage Engineering, College of Aburaihan, University of Tehran, Tehran, Iran; 4 Department of Water Engineering, Faculty of Agriculture, University of Tabriz, Tabriz, Iran; Sichuan University, CHINA

## Abstract

Simplified prediction of the interactions of plant tissue culture media components is of critical importance to efficient development and optimization of new media. We applied two algorithms, gene expression programming (GEP) and M5’ model tree, to predict the effects of media components on in vitro proliferation rate (PR), shoot length (SL), shoot tip necrosis (STN), vitrification (Vitri) and quality index (QI) in pear rootstocks (Pyrodwarf and OHF 69). In order to optimize the selected prediction models, as well as achieving a precise multi-optimization method, multi-objective evolutionary optimization algorithms using genetic algorithm (GA) and particle swarm optimization (PSO) techniques were compared to the mono-objective GA optimization technique. A Gamma test (GT) was used to find the most important determinant input for optimizing each output factor. GEP had a higher prediction accuracy than M5’ model tree. GT results showed that BA (Γ = 4.0178), Mesos (Γ = 0.5482), Mesos (Γ = 184.0100), Micros (Γ = 136.6100) and Mesos (Γ = 1.1146), for PR, SL, STN, Vitri and QI respectively, were the most important factors in culturing OHF 69, while for Pyrodwarf culture, BA (Γ = 10.2920), Micros (Γ = 0.7874), NH_4_NO_3_ (Γ = 166.410), KNO_3_ (Γ = 168.4400), and Mesos (Γ = 1.4860) were the most important influences on PR, SL, STN, Vitri and QI respectively. The PSO optimized GEP models produced the best outputs for both rootstocks.

## Introduction

Plant tissue culture technology has been extensively used for large-scale rapid crops multiplication. Besides the use of this technique as a tool of research, plant tissue culture has in recent decades, become of prime industrial significance in the area of propagating crops, eliminating disease, ameliorating house hold nutritional security, crop improvement and production of secondary plant metabolites [1]. Rootstocks play an important role in determining the final efficiency in orchard systems. The effect of rootstocks on precocity, crop yield, tree size control and fruit quality has been well demonstrated [2].

Pyrodwarf (‘Old Home’ × ‘Gute Luise’) and OHF 69 (‘Old Home’ × ‘Farmingdale’) are two well-known pear rootstocks with tolerance to winter cold, calcareous soils and fire blight. These rootstocks are compatible with all pear cultivars and are recommended for high-density planting systems [3–5].

Implementation of plant tissue culture for propagation of fruit trees has increased considerably since the early 1970s and allows breeders to rapidly propagate large numbers of new rootstocks [6]. The response of Pyrus species to in vitro culture varies significantly. Shoot tip necrosis [7–9], hyperhydricity [10–13], fasciation [14] and hooked leaves [15] are physiological disorders detected in some cases but successful tissue culture of this genus has also been reported [16].

Mineral and hormone composition of a plant micropropagation medium are essential factors in explant growth [17, 18]. Plant species differ in nutrient and hormone requirements, leading to development of numerous media formulations. However, optimizing or modifying media for a particular plant is a problematic and time consuming process which is usually done based on the history of current or previously used culture media for similar species and tissue culture systems [19, 20]. Choosing a medium based on prior usage may not give the same result response in other plant species [21, 22]. Improper mineral or hormone concentrations can cause growth inhibition or various physiological disorders including leaf chlorosis, shoot tip necrosis, hyperhydricity, and leaf spots [10, 17, 18]. Understanding the effects of minerals, hormones, and their interaction with other media ingredients and cultured tissues, is essential for successful in vitro plant propagation [17, 18].

Due to the complexity of interactions between media components, determining an optimized culture medium for a particular plant species or genotype is very difficult. Developing and implementing a reliable predictive modeling system could significantly improve the efficiency of this process [10, 18]. Due to their non-linear nature and complexities, modeling of biological systems is poorly understood [20]. Recently developed meta-modeling techniques appear to be promising methods for modeling complicated non-linear systems. The best examples are: genetic expression programming (GEP) [23–25], Artificial Neural Networks (ANN) [26, 27], Fuzzy Logic (FL) [28, 29] and statistical methodologies [15, 30].

Gago et al. [20] compared ANN with common statistical analysis and found ANN to be an efficient alternative for reliable evaluation of plant processes. Neurofuzzy logic was useful for optimizing nutrient and growth regulator concentrations in designing a new apricot micropropagation medium [31]. Neurofuzzy logic was also used to investigate the effects of light and sucrose on in vitro cultivation of kiwifruit [32].

Recently we successfully used a hybrid method, combining ANN with a genetic algorithm (GA), called ANN-GA, to model and predict an optimum culture medium composition for in vitro proliferation of G × N15 *Prunus* rootstocks [11]. When compared, the ANN-GA models were superior to traditional regression analysis [10].

Although previous studies indicated modeling methods based on Artificial Intelligence (AI) have high predictive accuracy and are more useful than other techniques [33–36], they are “black box” tools that do not provide a clear mathematical model based on input of independent variables.

The GEP model is another AI-based optimization method introduced by Ferreira [37] which includes beneficial attributes of both GA and genetic programming (GP). This new model based on an algorithm of evolving computer programs has been applied to diverse engineering problems and shown to accurately detect nonlinear and complex relations between input and output [23–25, 38]. Despite the potential benefits, no study has yet examined use of this method in the field of plant micropropagation.

Here, GEP is compared to M5’ model tree, a decision-tree-based algorithm for addressing problems and predicting output parameters. In M5’ model tree, data is divided into groups according to the most important input variables, and for each groups a multivariate linear regression equation is generated to evaluate the output variable. Advantages of the M5’ tree include simplicity, accuracy, and wide applicability [23, 25, 39] but this approach has not been used previously for plant micropropagation research.

Recently, in a case study [40], we compared use of Radial Basis Function Neural Network (RBFNN) to GEP for optimizing the composition of pear rootstock tissue culture media. We found GEP was a significantly robust and more accurate method than RBFNN for predicting proliferation quality and quantity. Therefore, a GA procedure was used to optimize GEP models [40]. However, GA could only optimize the level of inputs needed for each individual output separately and therefore could not provide a comprehensive optimum formulation for an entire culture medium.

Accordingly, the ability of multi-objective evolutionary algorithms (MOEAs) to lead us to an overall optimized media composition was assessed in the current research. With this method, inputs are assessed as multi-objective optimization problems (MOPs) and the solutions indicate the best possible balance between two opposite functions. This provides a solution set named the Pareto optimal set [41], and value of the corresponding objective function values procedure the Pareto front. In recent years, numerous mathematical techniques have been applied to solve MOPs, but the actual applications of MOPs are particularly nonlinear and also periodically non-differentiable [42]. This has boosted interest in metaheuristic approaches, and among these methods, MOEAs are of particular interest.

In recent decades, various MOEAs have been introduced for different population-based meta-heuristic algorithms, such as immune clone algorithm [43], GA [44], firefly algorithm [45] and PSO algorithm [46]. These types of algorithms generate a group of non-dominated solutions (also identified as Pareto-optimal solutions) where any improvement in one criterion constantly impairs other criteria. Additionally, these algorithms have a noticeable advantage; that is, population based, resulting generation of several Pareto optimal set [41] elements in a particular run, while the mathematical models make one element per run. The success of MOEAs in finding the best solution for MOPs suggested using these algorithms in this study. Here, two effective MOEAs, i.e. multi-objective GA (MOGA) and multi-objective particle swarm optimization (MOPSO), an evolutionary computation technique [47, 48], were compared for determining optimized culture media. Furthermore, we compared the results of the preferred MOP technique to the previously used mono-objective GA (MNOGA) optimization technique [40] to find the best method.

Despite various studies attempting to predict and model plant tissue culture media using nonlinear methods such as ANN [10, 11, 18, 49], there are still unanswered questions. The most important is how many data points are needed to achieve sufficiently accurate prediction? Which inputs are relevant or irrelevant in making a prediction model?

**Fig 1:**
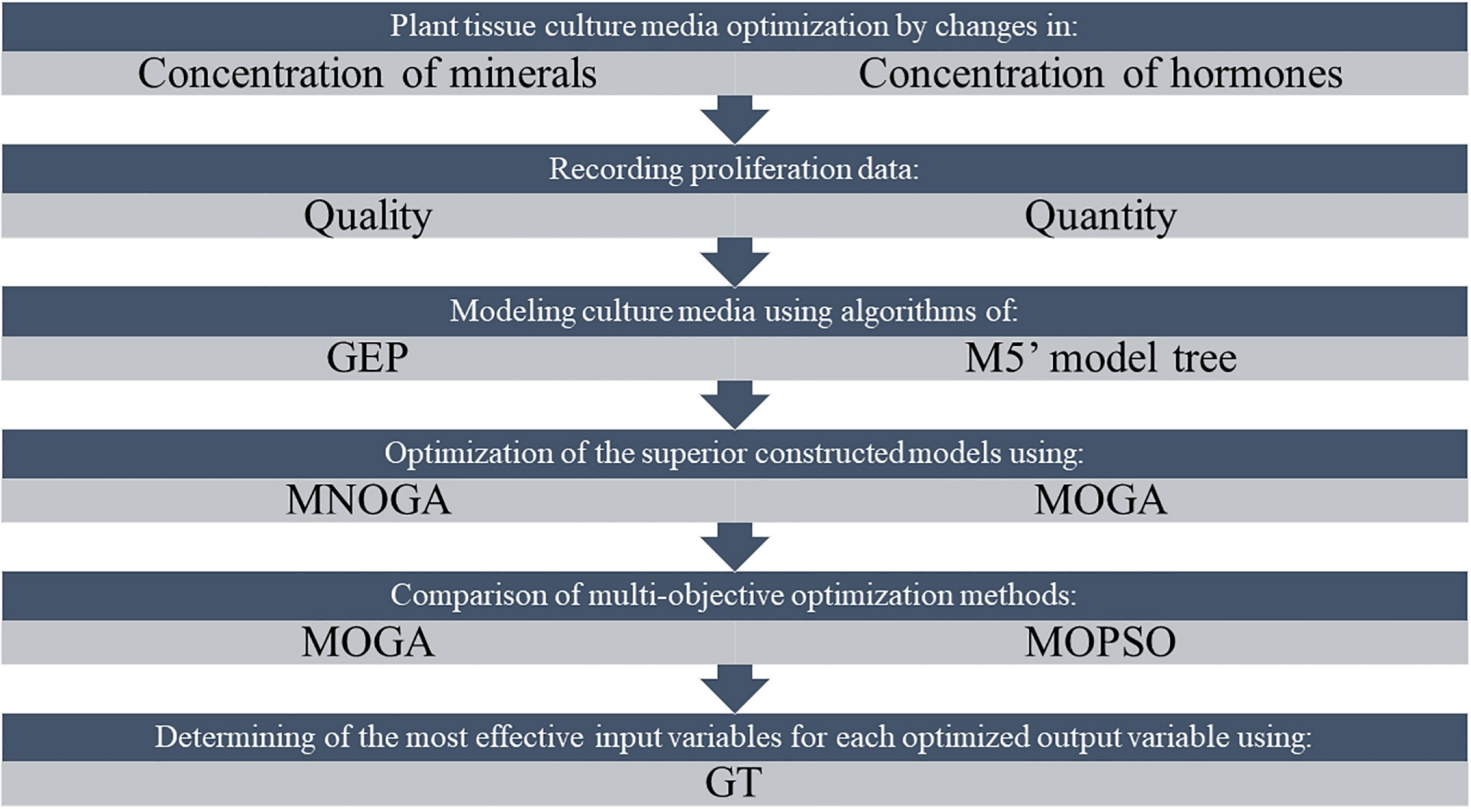
Framework of modeling and optimizing of pear rootstock in vitro proliferation media.

Recent computational technologies such as gamma test (GT), a novel algorithm from the computing science community, can now assist in solving these problems [50] and formal proofs of this method have been published [51–54]. This technique can help find the most appropriate input combinations to achieve a given targeted output. GT is also designed to solve the overtraining problem related to nonlinear modeling techniques such as ANN, by estimating how closely any smooth model fits the test data [52]. It has been demonstrated that GT can provide information about the relationship between input and output data sets, even before development of a model [55, 56]. Here, GT has been applied to select the most effective predicting input variable for each optimized output. A framework of the present study has been shown in Fig 1.

In brief, the new contributions of this research are:

- Evaluating the suitability of GEP and M5’ model tree nonlinear techniques for modeling plant tissue culture media development.
- Comparing the optimal solutions of MNOGA, MOGA and MOPSO in order to acquire a set of non-dominated solutions that are precise and efficient for the MOPs.
- Applying GT to assess the effect of minerals and hormones, in different combinations, on parameters of pear rootstock growth in vitro and to efficiently predict the effect of input factors.
- Exploring the role of culture media hormones, micro, and macro nutrients on in vitro performance of pear rootstocks.

To our knowledge, this is the first application of GEP, M5’ model tree, MOPSO, MOGA and GT techniques for optimizing plant tissue culture media.

## Material and methods

### Case studies

*Pyrus* rootstocks, Pyrodwarf and OHF 69 were grown on ^17^(MS) medium with modified mineral nutrients and supplemented with 30 g/l sucrose, 1 g/l myo-inositol, 8 g/l agar (DuchefaH), and various concentrations of BA and IBA. Media were adjusted to pH 5.7, poured into 250 ml jam jars with plastic caps, and autoclaved at 1 kg cm^-2^ s^-1^ (121 °C) for 15 min. All cultures were incubated in a growth chamber at 25 ± 2 °C under 16 h warm white fluorescent light (80 μmol m^-2^s^-1^) for 4 weeks. Afterwards, plant parameters PR, SL, Vitri, STN and QI, were recorded. For all experiments, each treatment consisted of 10 replicates (jam jars) containing four explants each for both Pyrodwarf and OHF rootstocks.

### Box–Behnken design as an invaluable tool for optimization of explant growth parameters

Box–Behnken Design (BBD) was used to statistically develop the model and to evaluate the main interactions and quadratic effects of six factors (KNO_3_, NH_4_NO_3_, mesos, micro-nutrients, BA and IBA) on the PR, SL, STN, Vitri and QI. BBD is a spherical, revolving response surface methodology (RSM) that consists of a central point and with the middle points of the edges of the cube circumscribed on the sphere. It consists of three interlocking 2^2^ factorial designs with points lying on the surface of a sphere surrounding the center of the design [57]. This design is more efficient and economical than their corresponding 3^k^ designs (k is factor number), mainly for a large number of variables [58–60]. The experimental ranges selected for independent variables are shown in Table 1. Each variable (Table 2) varied over a concentration range expressed in relation to MS medium (1× = MS concentration). After selection of independent variables and their ranges, the experiments were established based on a BBD with six factors at three levels and each independent variable was coded at three levels between +1, 0 and −1 corresponding to the low, mid and high levels (Table 3) [61]. The actual values and observed results for the three levels of the factors studied are presented in S1 and S2 Tables. Each mineral or hormone concentration treatment consisted of at least 10 replicates of four explants each.

**Table 1:** The factor components, range of experimental runs, and concentration ranges expressed as ×MS levels.

Factors	components	Range
Factor 1	KNO_3_	0.5–2 ×
Factor 2	NH_4_NO_3_	0.5–2 ×
Factor 3 (mesos)	CaCl_2_	0.5–2.5 ×
	KH_2_PO_4_	
	MgSO_4_	
Factor 4 (minors)	CoCl_2_.6H_2_O	0.5–4 ×
	CuSO_4_.5H_2_O	
	H_3_BO_3_	
	Kl	
	MnSO_4_.H_2_O	
	Na_2_MoO_4_.2H_2_O	
	ZnSO_4_.7H_2_O	
	FeNaEDTA	
Factor 5 (hormone)	BA	0.5–2.5 mgl^-1^
Factor 6 (hormone)	IBA	0.05–0.2 mgl^-1^

**Table 2:** The level of variables chosen for the Box–Behnken design.

Variable	Coded variable level		
	Low	Mid	High
	-1	0	1
**KNO**_**3**_ **(× MS)**	0.5	1.25	2
**NH**_**4**_**NO**_**3**_ **(× MS)**	0.5	1.25	2
**Mesos (× MS)**	0.5	1.5	2.5
**Minors (× MS)**	0.5	2.25	4
**BA (mg/l)**	0.5	1.75	3
**IBA (mg/l)**	0.05	0.13	0.2

**Table 3:** Coded factor levels for a Box-Behnken design of a six-variable system.

Culture medium	Level of factors in a coded form					
	KNO_3_	NH_4_NO_3_	Mesos	Minors	BA	IBA
1	0	-1	0	0	-1	1
2	1	1	0	-1	0	0
3	0	-1	1	0	-1	0
4	1	0	-1	0	0	-1
5	1	0	1	0	0	-1
6	1	0	0	-1	1	0
7	0	-1	0	0	1	-1
8	0	0	-1	1	0	-1
9	1	0	1	0	0	1
10	0	1	0	0	1	1
11	0	0	1	1	0	1
12	-1	-1	0	1	0	0
13	1	-1	0	1	0	0
14	0	1	-1	0	1	0
15	-1	0	-1	0	0	-1
16	1	-1	0	-1	0	0
17	0	-1	0	0	1	1
18	0	0	1	-1	0	1
19	0	-1	0	0	-1	-1
20	0	1	1	0	1	0
21	0	-1	-1	0	-1	0
22	1	0	-1	0	0	1
23	0	1	-1	0	-1	0
24	0	-1	-1	0	1	0
25	0	1	0	0	1	-1
26	-1	-1	0	-1	0	0
27	-1	0	0	1	-1	0
28	-1	0	1	0	0	1
29	1	0	0	1	1	0
30	0	0	-1	-1	0	1
31	-1	1	0	-1	0	0
32	0	0	1	-1	0	-1
33	-1	0	0	-1	1	0
34	0	0	1	1	0	-1
35	1	0	0	1	-1	0
36	0	0	-1	-1	0	-1
37	1	0	0	-1	-1	0
38	0	1	0	0	-1	1
39	0	1	0	0	-1	-1
40	-1	0	1	0	0	-1
41	1	1	0	1	0	0
42	0	-1	1	0	1	0
43	0	0	-1	1	0	1
44	-1	0	0	1	1	0
45	0	1	1	0	-1	0
46	-1	0	-1	0	0	1
47	-1	1	0	1	0	0
48	-1	0	0	-1	-1	0

### Implementation of the models

#### Gene expression programming (GEP)

**Fig 2:**
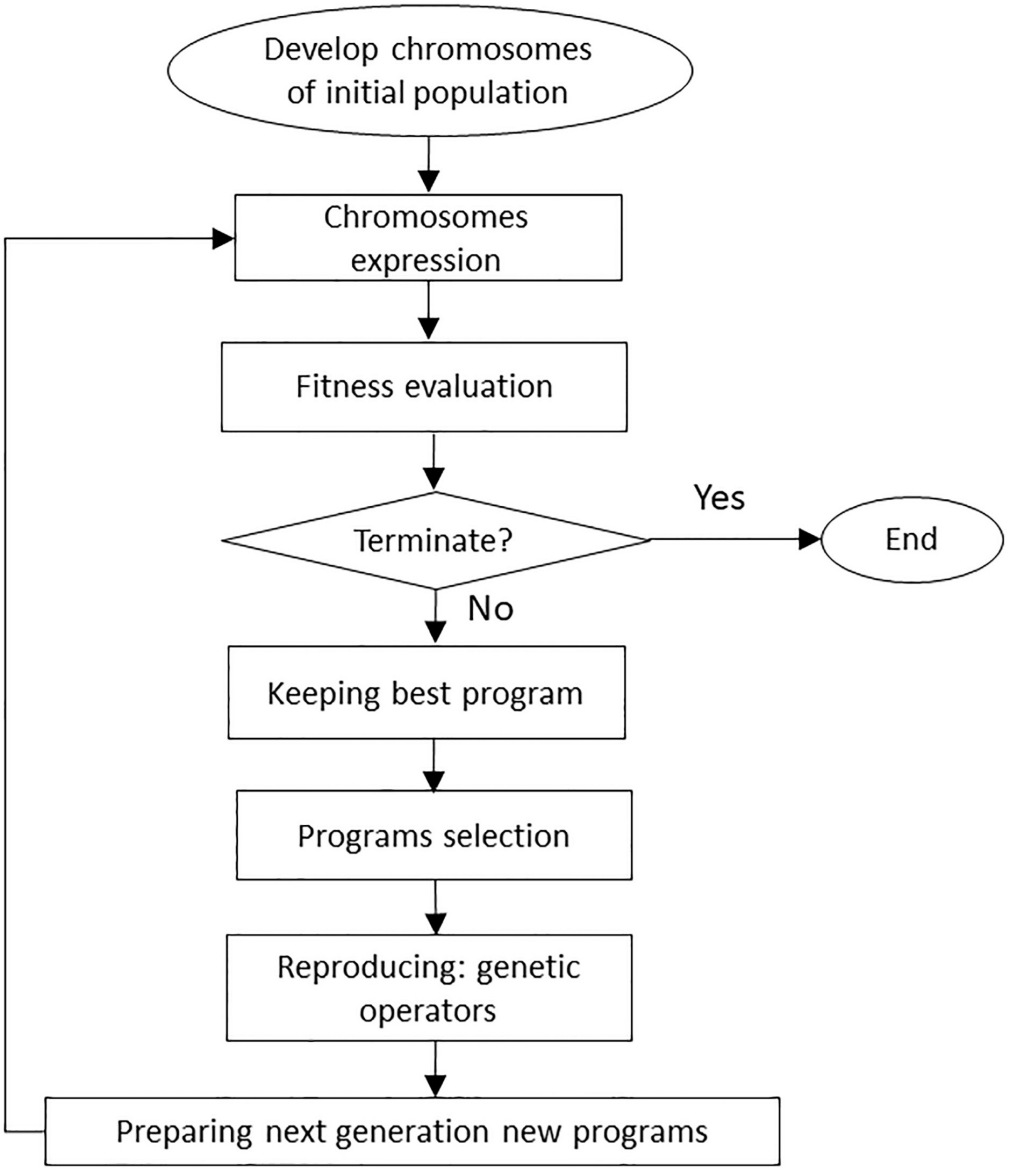
General structure and operation of the GEP model [66].

GEP, which was introduced by Ferreira [37] for the first time, is an evolutionary algorithm that evolves computer programs and predicts math models of experimental data. The algorithm of this method is similar to the GP method except that in this method fixed-length character strings called chromosomes are used to provide computer programs which are then expressed as expression trees [37, 62]. The structure of the linear chromosomes facilitates function of important genetic operators such as mutation, transposition and recombination. One of the advantages of the GEP approach is that search operators in this model produce valid structures and simplify creation of genetic diversity. The unique and multi-genic nature of this method allows very complex programs to be evolved [25, 62]. GEP is 100 to 60,000 times faster than the GP method [63]. There are several basic steps for preparation of this model. In the GEP algorithm, the process begins primarily with random production of chromosomes from a given number of individuals (the initial population of solutions). The chromosomes are shown as tree expression; in the next step, the performance or compatibility of each member of the population of the chromosomes should be evaluated. This is performed using a fitness function by which the fitness level of each individual is evaluated and selected based on its performance, to be bred, and a generation with new characteristics is formed; the generation produced is developed again to find a good and suitable solution. The third step comprises selection of a set of terminals and a set of functions for creating chromosomal genes. The set of terminals in the present study, including variables PR, SL, STN, Vitri and QI, with the selected functions for each GEP model, are presented in Table 4. The fourth step is to determine some of the control parameters of program running. These parameters, involving gene number, head length of each gene in chromosome, and genetic operators such as mutation, inversion, transportation, recombination, crossover, and one-point, two-point and gene recombination, are summarized in Table 4 [62]. The fifth step is to select the linking function. For algebraic sub-trees, the addition or multiplication linking function must be selected [37]. Generally, selection of the linking function depends on the nature of the problem and there is no basic rule for determining which of these functions is more appropriate [64]. Fig 2 shows the general structure of the GEP modeling method. In the present study, an additional linking function was used to create a link between sub-trees. Here, different GEP models were evaluated using GeneXpro package software [62].

**Table 4:** Training parameters of the GEP model.

Function set	+, -, ×, ÷ √, ∛, *sin*, *cos*, *Arctgx*, *x*^2, *x*^3, *e*^*x*, *ln*, Inverse, Tanh, Avg 2 inputs
Chromosomes	50
Head size	8
Number of genes	3
Linking functions	Addition
Fitness function error type	Root relative square error (RRSE)
Mutation rate	0.044
Inversion rate	0.1
One-point recombination rate	0.1
Two-point recombination rate	0.3
Gene recombination rate	0.1
Gene transportation rate	0.1

The GEP model is generally recommended and appropriate under the following conditions:

- Understanding the relationship among related variables is weak.
- Finding the final answer is difficult.
- Normal mathematical methods are not capable of analyzing the problem.
- Approximate answers are acceptable.
- There is a large amount of information that needs to be integrated, categorized and tested.
- Any minor improvement in the final answer is measurable and valuable [65, 66].

#### M5’ model tree

The M5’ model tree originally developed by Quinlan [67] is a binary decision tree with linear regression functions in the end nodes. These functions can make relationships between independent and dependent variables and predict continuous numerical characteristics. Generation production in this model involves two steps. The first step uses a separation index to create a decision tree. The separation index for the M5’ model tree algorithm is based on evaluation of the standard deviation of the class values devoted to each node. Standard deviation is used as a measure of the error in that node, which calculates the expected reduction in this error as a result of testing each attribute in that node. The formula for standard deviation reduction (SDR) is defined as follows:

$$SDR=sd(T)-\sum \frac{\left|T_{i}\right|}{\left|T\right|}sd(T_{i})$$ (1)

Where *T* represents a set of samples assigned to each node, *Ti* represents a subset of samples that has the *i*th output of a potential set, and *sd* is standard deviation [68].

Due to the process of separation and splitting, the standard deviation of the data in the progeny nodes (lower nodes) is lower than the value in the parent node [69]. After examining all possible separations, a separation that reduces expected standard deviation to its maximum value is selected. However, this categorization often leads to a large tree structure that is likely to result in over-training or weakness in the of model generalization. To solve this problem, in the second stage, the created tree is pruned and then the modified sub-branches are replaced by linear regression functions. This technique increases the accuracy of the tree model [67].

**Fig 3:**
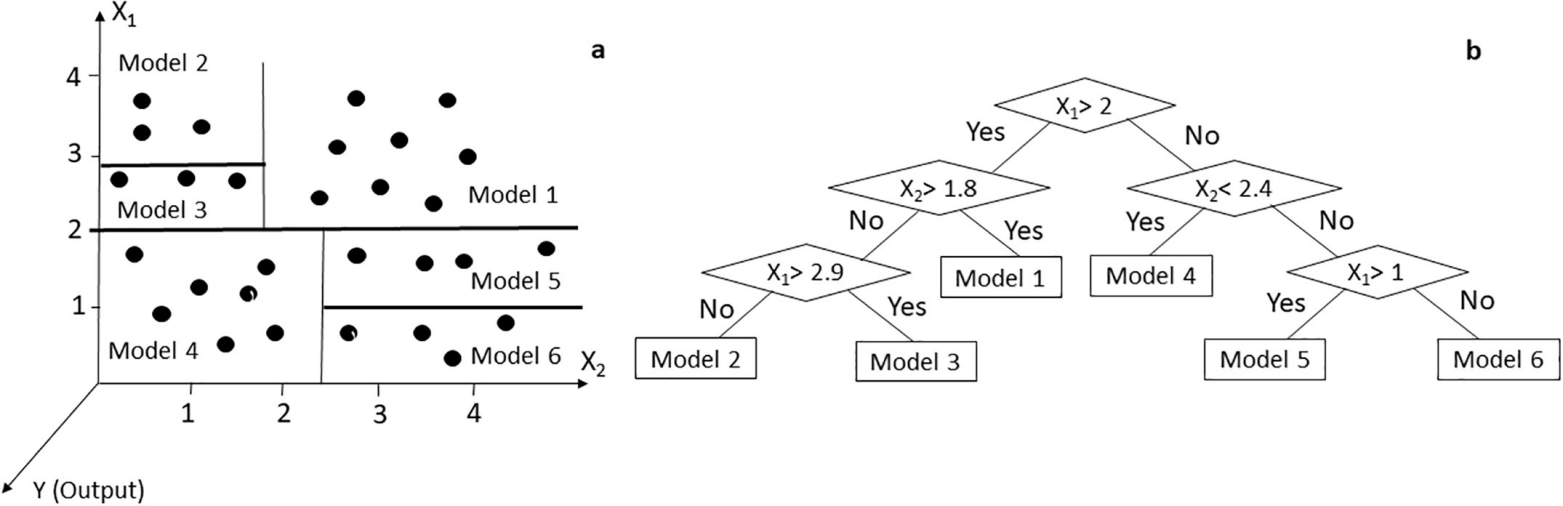
Separation of input space X_1_ × X_2_ by M5 model tree algorithm (a). Model tree diagram with six regression equations in leaves (b).

The M5’ model tree algorithm divides the parametric space into subspaces and generates a regression model in each of them. Fig 3a shows the separation of input space X1×X2 (independent variables) into six subspaces (leaves). In each of the leaves, a linear regression function called model 1 to model 6 are created. In Fig 3b these relationships are represented as a tree diagram, each of which is a leaf.

#### Gamma test-factor selection

GT is a nonlinear analysis and modeling tool that can examine the relationship between input and output of a numerical data set. In this method, based on input data, that part of the output variance that cannot be justified by smooth models is computed; although the model is unknown. The most important advantage of this tool is its speed of processing, even in the case of large data sets [70].

Assuming that we have a set of M member of the observational data, {(x_i_, y_i_): 1 ≤ i ≤ M}, then the relation between the input (x) and its corresponding output (y) can be expressed as follows:

$$\text{y}=\text{f}({\text{x}}_{1}\ldots .{\text{x}}_{\text{M}})+\varepsilon$$ (2)

Where f is a smooth function for data modeling and ε is a random variable representing an error. The GT method is based on the fact that if the two points x_1_ and x_2_ are in the vicinity of each other in the input space, then the corresponding outputs y_1_ and y_2_ must also be in the vicinity of each other in the output space. If these two outputs are not in the vicinity of each other, this difference is considered as an error. Estimates of this error by the delta function for input vectors and the gamma function for the output vector are as follows.

$$\delta_{\text{M}}(\text{k})=\frac{1}{\text{M}}\sum_{\text{i}=1}^{\text{M}}{\left|{\text{x}}_{\text{L}[\text{I},\text{K}]}-{\text{x}}_{\text{i}}\right|}^{2}(1\leq \text{k}\leq \text{p})$$ (3)

where |.| represents the Euclidean distance, -k x_L[i,k]_ is the closest neighbor for each vector (1 ≤ i ≤ M), and p is the number of close neighbors.

$$\gamma_{\text{M}}(\text{k})=\frac{1}{2\text{M}}\sum_{\text{i}=1}^{\text{M}}{\left|{\text{y}}_{\text{L}[\text{I},\text{K}]}-{\text{y}}_{\text{i}}\right|}^{2}(1\leq \text{k}\leq \text{p})$$ (4)

Where Y_L[i,k]_ denotes the corresponding values of y for -k, the nearest neighbor of the vector x_i_ in Eq 2. To calculate the amount of gamma statistics (Γ) by creating a linear regression based on the method of minimum number of squares among p pairs (δ_M_(k), γ_M_(k)), an equation is obtained as follows:

γ=Αδ+Γ (5)

The intercept of Eq 4 indicates the amount of gamma statistics (Γ). The small values of Γ indicate that the output can easily be predicted by the inputs. While the existence of large values of Γ indicates that there is no strong relationship between the selected inputs with the observed output, or some of the important variables are neglected or the number of input data used is low [51]. The slope of Eq 4 is an index of the complexity of the model. The higher the slope, the more sophisticated the system is [55]. Therefore, using the GT, the order of the importance of the input variables can be determined. To determine the order of the importance of the input parameters, in a step-by-step process, the GT is first performed for the complete composition (all input parameters). In the second step, one of the input parameters is arbitrarily deduced from the original compound and the Γ statistic is recalculated. In the third step, by returning the deleted parameter to the original compound, another variable is deleted, and then the GT is executed for the new composition. This process is repeated for all parameters. Finally, the parameter which, with its removal from the original composition, least increases the Γ statistic, is least important, and the parameter which, with its removal from the original composition, has the highest Γ statistic, is the most important variable affecting the desired output.

### Optimization models

#### Genetic algorithm

GA is a renowned global optimization method according to the Darwin’s principle of the ‘survival of the fittest’ and the natural process of evolution by reproduction. GA keeps a set of nominee solutions called “population” and repetitively modifies them. At each stage, the GA chooses individuals from the present population as parents and applies them to create the children for the next generation. Nominee solutions are typically represented as strings of fixed length, called chromosomes. A fitness or objective function is applied to reflect the appropriateness of each population member [71]. A specified random initial population GA runs in cycles called generations, as follows [72]:

- Fitness function is used to assess each member of the population.
- In a number of repetitions, the population is reproduced. One or more parents are selected randomly, however strings comprising higher fitness value have higher possibility of contributing an offspring.
- Genetic operators, like crossover and mutation are used to parents to create offspring.
- The offspring are placed in the population and the process is reiterated.

Application of GA requires determination of six basic items: chromosome representation, the genetic operators, selection function, initialization, termination and evaluation function. Various demonstrations of an individual or chromosome are: floating point numbers, binary digits, real values, integers, matrices, etc. Likewise, there are numerous patterns for the selection process: scaling techniques, roulette wheel selection and its extensions, normal geometric, tournament, elitist models and ranking methods. There are two fundamental kinds of genetic operators; crossover and mutation. Crossover gets two individuals and creates two new individuals while mutation changes one individual to produce a distinct new solution. The following genetic operators are usually used: uniform mutation, non-uniform mutation, multi-non-uniform mutation, boundary mutation and simple crossover, arithmetic crossover and heuristic crossover. Moreover, random initialization and specified generations are commonly applied for the initialization and termination process [71].

#### Particle swarm optimization model

PSO is an evolutionary calculation method and swarm intelligence algorithm based on population to solve the inclusive optimization problem that was created by Eberhart and Kennedy [47]. It is a mathematical computation technique which starts with a crowd of grain named as the swarm and generally according to social models, like bird flocking, fish schooling and the swarm theory [40]. The main factors of PSO are the concomitant of swarm’s behavior i.e. retaining optimal distances between individual members and their neighbors. For optimizing the location of each particle, their position is improved as planned for the objective function inside the search region. So, the velocity of a particle is the main factor of PSO that is optimized in each repetition by comparing to the previous one to direct the particle to its best location. In each repetition, every particle in a swarm attains the best solution (fitness) up to now, named pbest. Additional “best” value which is a particle is acquired so far in the population followed by the particle swarm optimizer that is global best and named gbest. The velocity of every particle in a swarm is calculated through the following equation [47, 48].

$$V_{i+1}=wV_{i}+c_{1}r_{1}(p{Best}_{i}-X_{i})+c_{2}r_{2}({gBest}_{i}-X_{i})$$ (6)

$$X_{i+1}=X_{i}+V_{i+1}$$ (7)

where, *V*_*i+1*_ is new velocity for every particle according to previous velocity (*Vi*), W is inertial coefficient (0.8–1.2), c_1_ and c_2_ is cognitive coefficient and social coefficient, respectively (0–2), *r*_*1*_ and *r*_*2*_ is random values for every velocity update (0–1) and *X*_*i+1*_ is new position for every particle based on the previous position (*Xi*).

#### Performance evaluation of the proposed models

In order to investigate and compare the accuracy of different models, four statistical measures; root mean square error (RMSE), mean absolute relative error (MARE), mean bias error (MBE) and correlation coefficient (R), were used. Their equations are as follows:

$$RMSE=\sqrt{\frac{\sum_{i=1}^{N}{\left(O_{i}-P_{i}\right)}^{2}}{N}}$$ (8)

$$MARE=\frac{1}{N}\sum_{i=1}^{N}\left|\frac{O_{i}-P_{i}}{O_{i}}\right|$$ (9)

$$MBE=\frac{1}{N}\sum_{i=1}^{N}\left(O_{i}-P_{i}\right)$$ (10)

$$R=\frac{\sum_{i=1}^{N}\left(O_{i}-{\overset{-}{O}}_{i}\right)(P_{i}-{\overset{-}{P}}_{i})}{\sqrt{\sum_{i=1}^{N}{\left(O_{i}-{\overset{-}{O}}_{i}\right)}^{2}\sum_{i=1}^{N}{\left(P_{i}-{\overset{-}{P}}_{i}\right)}^{2}}}$$ (11)

Where *O*_*i*_ and *P*_*i*_ are the observed and predicted values, respectively and ${\overset{-}{O}}_{i}$ and ${\overset{-}{P}}_{i}$ are the mean observational and the predicted values for N number of samples. Parameters are analyzed together to attain a precise medium composition.

## Results

### Performances of modeling procedures

Our models of the effects of altering inputs were constructed using M5’ model tree and GEP techniques. The GEP predictive model explained variation in most of the growth parameters, the Equations that best estimated these parameters are in Table 5. A second set of equations was established using M5’ model trees (Table 6).

**Table 5:** Equations developed using gene expression programming for predicting explant growth parameters.

Pear Rootstock	Equation
**Pyrodwarf**	$PR=[\text{exp}((F-B)\times c)-D]+[{\left(\text{ln}\left(\frac{\sqrt{\frac{E-2.1016}{2}}+E}{2}\right)\right]}^{-1}+D)+[{\left(\sqrt[{3}]{3.1775-C}\right)}^{2}+\sqrt[{3}]{\frac{\text{exp}\left(A\right)}{C+0.11065}}]$
	$SL=[\sqrt[{3}]{\text{ln}(D^{3})}+(F\times (\frac{C+A}{2}\times E))]+[2.4011\times {\left(\frac{\left(\frac{E+1.7251}{2}\times 2.7057)+(1.7251-B\right)}{2}-E\right)}^{2}]+[\sqrt[{3}]{2.3488\times (\left(E^{-1}-7.0907)+(E-D\right))}]$
	$STN=[\frac{D+(\sqrt{C}\times (F-E))}{2}+exp(A-C)]+[\left(B-\frac{\frac{6.1753}{E}+E}{2})+(9.3446A\times \text{ln}(B)\right)]+[\frac{((\text{ln}\left(F\right)+\frac{11.3038}{A})-E+1.0814)+15.0404A^{2}}{2}]$
	$VITRI=[\frac{(\text{exp}(B)\times B)\times {\text{C}}^{-1}}{2}-(D-B)-1.5069]+[\frac{\text{exp}\left(2.988-D\right)+\frac{A-9.0917}{2}}{2}\times (2B-(B\times D))]+[\frac{\frac{\frac{E+B}{2}+B}{2}+F+8.8145}{2}\times (\frac{A+B}{2}+2B)]$
	$QL=[\sqrt[{9}]{\frac{D\times A}{\sqrt{C}\times B}}]+[\sqrt[{3}]{{\left(\frac{\frac{E}{30.7869}+B}{2}\right)}^{-1}+(0.9692-A-B)}]+[\sqrt[{6}]{(\text{exp}\left(C\right)\times (10.3785+F))+F^{-1}-A}]$
**OHF**	
	$PR=[\sqrt{(0.0894D\times B)+{\left(A+E\right)}^{-1}}]+[(\sqrt[{3}]{\frac{-8.5762}{A}-6.8234})\times {\left(3.2661-E\right)}^{-1}]+[\frac{\frac{C+0.1838}{2}+6.8196}{2}+C^{-1}+((B\times F)-B)]$
	$SL=[\frac{\text{ln}\left(\frac{B+4.8333}{2}\right)+(\frac{D+4.7651}{2}-(A+B))}{2}]+[C+(\text{exp}((-2.8335A)\times (\frac{\left(D-6.4679)+(9.3060C\right)}{2})))]+[\text{exp}(\frac{\left((3.8362F)-A)+(\frac{E+3.1237}{2}\right)}{2}-{\left(3.4882-E\right)}^{-1})]$
	$STN=[B^{2}-(\sqrt[{3}]{\frac{\frac{C}{E}+8.1303}{\text{ln}(A)}})]+[\text{exp}\left(A\right)+C^{3}-C]+[F+\frac{D+({\left(\frac{-4.4380}{C}\right)}^{2}-\frac{6.1907}{B})}{2}]$
	$VITRI=[E\times (A+\frac{(\frac{E-0.8805}{2})\times B}{F+A})]+[\frac{A-(\sqrt[{3}]{A}-6.8169)}{D^{2}}]+[\left(\frac{A+2D-6.8579}{2})\times (\frac{D^{2}+\frac{B}{A}}{2}\right)]$
	$QL=[\sqrt{{\left(\sqrt[{4}]{\left(\frac{E^{-1}+8.3712}{2})\times (C-F\right)}\right)}^{3}}]+{\left[\left((C-5.1079)\times \sqrt{D})\times ((C\times A)-5.0292\right)\right]}^{-1}+[\frac{B^{-1}+ln(\left(ln\frac{C}{0.3661})\times (D+F\right))}{2}]$

A: NH_4_NO_3_, B: KNO_3_, C: Mesos, D: Minor, E: BA, F: IBA.

**Table 6:** Rules of the M5ʾ tree model for OHF and Pyrodwarf in vitro proliferation parameters.

Pear rootstock	Growth parameter	Rule number	If	Then
**OHF**	**Proliferation rate**			
		1	E > 1.125	X = 0.4237 × A − 0.257 × B − 1.0822 × C + 0.0318 × D − 0.0738 × E + 0.7144 × F + 7.8457
			E <= 2.375	
			C > 1	
		2	E > 1.125	X = 0.3937 × A − 0.4698 × B − 1.2439 × C − 0.1651 × E + 0.1836 × F + 6.923
			E > 2.375	
		3	E <= 1.125	X = 0.1782 × A − 0.3381 × B − 0.9584 × C + 0.6468 × E + 1.2824 × F + 2.9123
			A > 0.875	
		4		X = 0.7912 × A − 6.2314 × C + 1.5998 × F + 9.8904
	**Shoot length**			
		1	C <= 2	X = -0.3552 × A − 0.2809 × B + 0.0958 × C + 0.0602 × D − 0.1355 × E + 2.4832 × F + 3.5306
			C > 1	
			D > 1.375	
			E > 2.375	
			B > 0.875	
		2	C > 1	X = -0.3463 × A − 0.0924 × B + 0.8286 × C + 0.0443 × D − 0.1156 × E + 3.1266 × F + 3.035
			D > 1.375	
			A > 0.875	
			E <= 2.375	
			A <= 1.625	
			B <= 1.625	
		3	C > 1	X = 0.1545 × A − 0.0727 × B + 0.5525 × C − 0.1703 × D − 0.3319 × E + 1.344 × F + 2.9199
			D > 1.375	
			A > 0.875	
			B > 0.875	
			F <= 0.163	
		4	C > 1	X = -0.0352 × A − 0.0992 × B + 0.2924 × C + 0.0807 × D − 0.1768 × E + 1.5625 × F + 3.2275
			D > 1.375	
			A > 0.875	
		5	C > 1	X = -0.0488 × A − 0.2705 × B + 0.3694 × C + 0.1452 × D + 0.0668 × E + 1.7824 × F + 3.2257
			D > 1.375	
			B > 0.875	
		6	A <= 1.625	X = -0.2058 × A − 0.3384 × B + 0.2668 × C + 0.2046 × D − 0.1517 × E + 1.8772 × F + 2.6147
			C <= 1	
			A > 0.875	
			E <= 2.375	
			D <= 3.125	
		7	A <= 1.625	X = -0.1354 × A − 0.3512 × B + 0.5812 × C + 0.2623 × D − 0.106 × E + 1.7793 × F + 2.4655
			E <= 2.375	
			C <= 2	
			B > 0.875	
			F <= 0.163	
		8	E <= 2.375	X = 0.2111 × A − 0.0768 × B + 0.5576 × C + 0.421 × D − 0.0584 × E + 1.2153 × F + 2.1238
			A <= 1.625	
			C > 1	
		9	E <= 2.375	X = -0.1813 × A − 0.2502 × B + 0.1778 × C − 0.0823 × E + 1.7182 × F + 2.8632
			A > 1.625	
			C <= 1	
		10	F <= 0.163	X = 0.1754 × A − 0.1856 × B − 0.0808 × E + 4.0154 × F + 2.0236
			E <= 2.375	
			B > 0.875	
		11	E > 2.375	X = 0.0641 × A − 0.1203 × B − 0.3621 × E + 3.2001
			B <= 1.625	
			A <= 1.625	
		12	E > 2.375	X = 0.4331 × A − 0.5811 × E + 3.0243
		13	A <= 1.625	X = 0.0481 × A + 3.2957
				X = + 3.245
	**Quality index**			
		1	C > 1	X = -0.2346 × A − 0.465 × B − 0.3995 × C − 0.373 × D − 0.1173 × E + 6.7724
			D > 1.375	
		2	C > 1	X = -0.4178 × A − 0.038 × B + 0.0916 × C − 0.1751 × E + 3.1081
		3	A <= 1.625	X = -0.0329 × A − 0.2049 × B+ 0.0499 × D- 0.0889 × E + 1.9649
			D > 1.375	
			B > 0.875	
		4		X = 0.3466 × A − 1.2761 × B + 0.14 × E − 1.0282 × F + 2.2133
	**Shoot tip necrosis**			
		1	C > 1	X = 2.355 × B + 5.6024 × C + 0.5965 × D − 1.7076
			B > 0.875	
		2	C > 1	X = 2.8713 × B − 1.658 × C + 0.1911 × D + 1.0358 × E + 66.0559 × F − 1.7365
			F <= 0.163	
		3	B > 0.875	X = 0.5473 × A + 0.862 × B − 6.8996 × C + 1.0135 × E + 9.4435 × F + 39.7179
			A <= 1.625	
		4	C <= 1	X = 13.3659 × A − 12.7662 × C − 1.4668 × E + 13.6242 × F + 24.3603
	**Vitrification**			
		1	D > 1.375	X = 4.1865 × A + 0.9834 × B − 0.6441 × C + 5.5661 × D + 2.1536 × E − 18.0036
		2		X = 4.544 × A + 2.8493 × E + 19.091
**Pyrodwarf**	**Proliferation rate**			
		1	E > 1.125	X = -1.8814 × A − 0.727 × B − 1.4415 × C + 0.0681 × D − 0.1405 × E + 1.6517 × F + 13.9309
			E <= 2.375	
			A <= 1.625	
			C <= 2	
		2	E > 1.125	X = -0.6304 × A − 0.2558 × B − 0.0147 × C + 0.0438 × D − 0.2349 × E + 0.3444 × F + 10.3964
			E <= 2.375	
			A <= 1.625	
		3	E > 1.125	X = -0.1087 × A − 0.2089 × B − 0.5264 × C − 0.0079 × D − 0.1992 × E + 1.6239 × F + 7.2824
			E > 2.375	
			B > 0.875	
			A <= 1.625	
		4	E > 1.125	X = -0.9459 × A − 0.2805 × B − 0.6259 × C − 0.0565 × D − 0.4487 × E + 0.3839 × F + 10.0108
			E > 2.375	
		5	E <= 1.125	X = -0.1673 × A − 0.3295 × B − 0.6127 × C + 0.0083 × D + 0.8668 × E + 1.3144 × F + 2.9653
			B <= 0.875	
		6	E > 1.125	X = -0.089 × A − 0.3747 × B − 0.2561 × C + 0.048 × D + 1.2633 × E + 1.1152 × F + 7.3119
			B <= 1.625	
			C <= 2	
		7	E > 1.125	X = -0.152 × A − 0.7885 × B − 0.0643 × C + 0.0796 × D + 2.0641 × E + 2.6466 × F + 5.5749
		8	A <= 0.875	X = -0.1996 × A − 0.2481 × C + 0.0398 × D + 2.0721
		9	A <= 1.625	X = -0.1075 × A − 0.3785 × C + 0.268 × F + 1.878
			C > 1	
		10		X = -1.3167 × A + 0.0357 × D + 3.6155
	**Shoot length**			
		1	C <= 2	X = -0.3552 × A − 0.2809 × B + 0.0958 × C + 0.0602 × D − 0.1355 × E + 2.4832 × F + 3.5306
			C > 1	
			D > 1.375	
			E > 2.375	
			B > 0.875	
		2	C > 1	X = -0.3463 × A − 0.0924 × B + 0.8286 × C + 0.0443 × D − 0.1156 × E + 3.1266 × F + 3.035
			D > 1.375	
			A > 0.875	
			E <= 2.375	
			A <= 1.625	
			B <= 1.625	
		3	C > 1	X = 0.1545 × A − 0.0727 × B + 0.5525 × C − 0.1703 × D − 0.3319 × E + 1.344 × F + 2.9199
			D > 1.375	
			A > 0.875	
			B > 0.875	
			F <= 0.163	
		4	C > 1	X = -0.0352 × A − 0.0992 × B + 0.2924 × C + 0.0807 × D − 0.1768 × E + 1.5625 × F + 3.2275
			D > 1.375	
			A > 0.875	
		5	C > 1	X = -0.0488 × A − 0.2705 × B + 0.3694 × C + 0.1452 × D + 0.0668 × E + 1.7824 × F + 3.2257
			D > 1.375	
			B > 0.875	
		6	A <= 1.625	X = -0.2058 × A − 0.3384 × B + 0.2668 × C + 0.2046 × D − 0.1517 × E + 1.8772 × F + 2.6147
			C <= 1	
			A > 0.875	
			E <= 2.375	
			D <= 3.125	
		7	A <= 1.625	X = -0.1354 × A − 0.3512 × B + 0.5812 × C + 0.2623 × D − 0.106 × E + 1.7793 × F + 2.4655
			E <= 2.375	
			C <= 2	
			B > 0.875	
			F <= 0.163	
		8	E <= 2.375	X = 0.2111 × A − 0.0768 × B + 0.5576 × C + 0.421 × D − 0.0584 × E + 1.2153 × F + 2.1238
			A <= 1.625	
			C > 1	
		9	E <= 2.375	X = -0.1813 × A − 0.2502 × B + 0.1778 × C − 0.0823 × E + 1.7182 × F + 2.8632
			A > 1.625	
			C <= 1	
		10	F <= 0.163	X = 0.1754 × A − 0.1856 × B − 0.0808 × E + 4.0154 × F + 2.0236
			E <= 2.375	
			B > 0.875	
		11	E > 2.375	X = 0.0641 × A − 0.1203 × B − 0.3621 × E + 3.2001
			B <= 1.625	
			A <= 1.625	
		12	E > 2.375	X = 0.4331 × A − 0.5811 × E + 3.0243
		13	A <= 1.625	X = 0.0481 × A + 3.2957
		14		X = + 3.245
	**Quality index**			
		1	C > 1	X = -0.2346 × A − 0.465 × B − 0.3995 × C − 0.373 × D − 0.1173 × E + 6.7724
			D > 1.375	
		2	C > 1	X = -0.4178 × A − 0.038 × B + 0.0916 × C − 0.1751 × E + 3.1081
		3	A <= 1.625	X = -0.0329 × A − 0.2049 × B + 0.0499 × D − 0.0889 × E + 1.9649
			D > 1.375	
			B > 0.875	
		4		X = 0.3466 × A − 1.2761 × B + 0.14 × E − 1.0282 × F + 2.2133
	**Shoot tip necrosis**		C > 1	X = 2.355 × B + 5.6024 × C + 0.5965 × D − 1.7076
			B > 0.875	
		1	C > 1	X = 2.8713 × B − 1.658 × C + 0.1911 × D + 1.0358 × E + 66.0559 × F − 1.7365
			F <= 0.163	
		2	B > 0.875	X = 0.5473 × A + 0.862 × B − 6.8996 × C + 1.0135 × E + 9.4435 × F + 39.7179
			A <= 1.625	
		3	C <= 1	X = 13.3659 × A − 12.7662 × C − 1.4668 × E + 13.6242 × F + 24.3603
	**Vitrification**			
		1	D > 1.375	X = 4.1865 × A + 0.9834 × B − 0.6441 × C + 5.5661 × D + 2.1536 × E − 18.0036
		2		X = 4.544 × A + 2.8493 × E + 19.091

Inputs are A: KNO_3_, B: NH_4_NO_3_, C: Mesos, D: Micros, E: BA, F: IBA.

The performance criteria values for the models are shown in Table 7. It is clear that both models reasonable predict all plant parameters measured. In comparing the accuracy, GEP was more precise for most parameters in both rootstocks except for shoot length. Consequently, GA and PSO techniques were used for multi-objective optimization of GEP models.

**Table 7:** Statistical summary of the implemented models.

Rootstock	Model	Output	RMSE	MARE	MBE	R^2^
**OHF**		Proliferation				
	M5		0.2756	0.0573	0.0442	0.9916
	GEP		0.2538	0.0599	0.0256	0.9933
**Pyrodwarf**						
	M5		0.3312	0.0512	-0.0782	0.9948
	GEP		0.3933	0.0708	0.0466	0.9932
**OHF**		Shoot length				
	M5		0.1665	0.0419	0.0024	0.9854
	GEP		0.2196	0.0587	0.0341	0.9741
**Pyrodwarf**						
	M5		0.1709	0.0921	-0.0795	0.9924
	GEP		0.2458	0.1258	0.0129	0.9783
**OHF**		Vitrification				
	M5		3.6278	0.1407	-0.0359	0.9497
	GEP		3.0738	0.1093	0.2496	0.9692
**Pyrodwarf**						
	M5		5.3214	0.1684	0.645	0.9135
	GEP		4.3844	0.1115	-0.8040	0.9364
**OHF**		Shoot tip necrosis				
	M5		4.1123	0.1735	0.453	0.9529
	GEP		4.0425	0.1319	-0.7604	0.9616
**Pyrodwarf**						
	M5		4.5173	0.1387	-0.4049	0.9327
	GEP		3.6175	0.1495	-0.2423	0.9544
**OHF**		Quality index				
	M5		0.3948	0.1191	-0.0098	0.934
	GEP		0.4067	0.1271	0.0717	0.9437
**Pyrodwarf**						
	M5		0.407	0.1428	-0.0109	0.933
	GEP		0.3782	0.1300	-0.0497	0.9440

### Optimization of GEP models

MNOGA and MOGA optimized GEP models were compared for their ability to optimize the composition in vitro culture media for pear rootstocks (Tables 8 and 9).

**Table 8:** Results of mono-objective optimization of a GEP model using GA to achieve maximum PR, SL and QI and minimum STN and Vitri during OHF and Pyrodwarf pear rootstock proliferation in vitro.

*Pyrus* rootstock		NH4NO3	KNO3	Mesos	Minor	BA	IBA
Pyrodwarf	PR						
	13.00432	1.5637	1.188	1.7548	3.2834	2.0792	0.1311
	SL						
	4.9999	1.8265	0.5115	1.7393	2.6042	0.5947	0.1599
	QI						
	4.9886	0.8855	0.5	2.5	3.9999	0.5001	0.05
	STN						
	0.0001	0.9254	0.5034	2.2966	0.6374	0.5129	0.0662
	Vitri						
	3.9989	0.5	0.5	2.4999	2.7638	0.5	0.05
OHF							
	PR						
	9.4209	1.9996	1.1895	0.6188	2.1306	1.5938	0.1721
	SL						
	4.9923	0.8990	1.3538	2.0435	3.4533	1.8080	0.0976
	QI						
	4.8365	0.9914	0.533	1.8578	2.1212	2.5860	0.1800
	STN						
	0.0185	1.0829	0.5144	1.5816	2.3630	1.9298	0.1504
	Vitri						
	0.0187	0.5	1.274	1.4925	1.5196	1.3158	0.1443

**Table 9:** Multi-objective optimization of GEP models using GA and PSO techniques to achieve the highest quality and quantity during pear rootstock in vitro proliferation.

Rootstock	Optimization technique	Medium composition						PR	SL	QI	STN	Vitri
		NH_4_NO_3_	KNO_3_	Mesos	Micro	BA	IBA					
**Pyrodwarf**	**PSO**	0.74	0.50	2.50	2.54	3.00	0.02	12.84	4.79	4.92	1.07	5.66
	**GA**	0.81	0.50	2.50	2.53	0.50	0.02	5.45	9.25	5.00	0.14	4.79
**OHF**	**PSO**	1.00	0.50	2.32	2.32	2.10	0.20	12.41	5.61	4.27	0.00	2.13
	**GA**	1.01	0.50	2.35	2.32	2.10	0.20	12.58	5.45	4.19	0.00	2.14

For Pyrodwarf, MOGA optimized models gave a lower PR (5.45) than MNOGA optimized models (13.00). On the contrary, SL was much lower in MNOGA (5.00) than MOGA (9.25) optimized models. QI showed almost no difference between the two procedures (4.99 vs.5.00). STN and Vitri were higher with MOGA optimization (0.14 and 4.79, respectively) than with MNOGA (0.0001 and 4.00) (Tables 8 and 9).

The same comparisons for OHF showed higher PR and SL with MOGA (12.58 and 5.45) than using MNOGA (9.42 and 4.99) but lower QI using MOGA (4.19) than MNOGA (4.84). STN was higher with MNOGA (0.02) than MOGA (0.00) and Vitri was much higher with MOGA (2.14) than MNOGA (0.02) (Tables 8 and 9).

MOPSO analysis on the GEP models resulted in optimal outputs of (mgl^-1^) 0.74 NH_4_NO_3_, 0.50 KNO_3_, 2.50 meso-nutrients (CaCl_2_, MgSO_4_ and KH_2_PO_4_), 2.54 micro-nutrients (CoCl_2_, CuSO_4_, FeNaEDTA, H_3_BO_3_, KI, MnSO_4_, NaMoO_4_ and ZnSO_4_), 3.00 BA and 0.02 IBA for Pyrodwarf and 1.00 NH_4_NO_3_, 0.50 KNO_3_, 2.32 meso-nutrients, 2.32 micro-nutrients, 2.10 BA and 0.20 IBA for OHF. In contrast, MOGA optimization analysis of GEP models gave medium containing (mgl^-1^) 0.81 NH_4_NO_3_, 0.50 KNO_3_, 2.50 meso-nutrients, 2.53 micro-nutrients, 0.50 BA and 0.02 IBA for Pyrodwarf and 1.01 NH_4_NO_3_, 0.50 KNO_3_, 2.35 meso-nutrients, 2.32 micro-nutrients, 2.10 BA and 0.20 IBA for OHF.

For Pyrodwarf, multi-objective optimizing of GEP models using PSO resulted in greater PR (12.84) than use of GA (5.45) but SL and QI were better optimized using GA than PSO (Tables 8 and 9). For OHF few differences in PR, Vitri, or STN were found between MOGA and MOPSO. The OHF, SL and QI were better optimized by MOPSO than MOGA, the opposite of the Pyrodwarf results (Tables 8 and 9).

The media constituents proposed by MOGA and MOPSO optimized GEP predictive models showed nearly the same input quantities for each component except BA hormone which was much greater in the MOPSO (3.00) than the MOGA (0.50) optimized model for Pyrodwarf rootstock.

### Important inputs selection using GT

For building GEP and M5’ models to predict culture media composition for pear rootstocks, key input factors were selected using the GT. Conventionally, a modeler needs to use trial and error to build mathematical models, such as ANN, to evaluate input combinations. This is very time consuming since the modeler needs to calibrate and test models using all the likely input combinations. In addition, there is no clear rule determining the number of data points to be used in the calibration process. Using the GT, the workload for model development can be reduced greatly and guidance can be provided regarding the number of input data used in the developed model. Basically, the GT helps reach the best possible mean square error that can be achieved using any nonlinear smooth model. In this study, various combinations of minerals and hormones were explored to assess their effect on explant growth of two pear rootstocks.

To determine the most important variables, the gamma value must first be calculated for a combination of all variables (6 input variables). In the next stage, one of the variables is omitted and the gamma value is calculated for a combination of the remaining variables (5 variables). Then, the omitted variable from the previous stage is returned and another variable is omitted from the original combination (6 variables) and a gamma value is then calculated for the new combination. This process is continued for all variables one by one and in each step the gamma value is computed. Thus, the inputs can be ranked based on the importance of their effect on the outputs using their gamma values.

For the OHF rootstock, BA, with the highest GT value (Γ = 4.0178), was identified as the most important input variable affecting the PR. Others in descending order were NH_4_NO_3_, Mesos, IBA, KNO_3_ and Micros (Table 10). Mesos (Γ = 0.5482) were the most significant factor affecting SL, followed by KNO_3_, IBA, BA, NH_4_NO_3_ and Micros. The relative importance of inputs affecting STN was Mesos (Γ = 184.0100) > IBA (Γ = 130.9600) > KNO_3_ (Γ = 127.3200) > NH_4_NO_3_ (Γ = 116.5100) > BA (Γ = 106.2700) > Micro (Γ = 44.7600). Micro (Γ = 136.6100), was the key variable influencing Vitri, while BA (Γ = -1.9649) and Mesos (Γ = -0.0819) had little effect. Mesos (Γ = 1.1146) were the greatest influence on QI. In general, many of the parameters for OHF were significantly influenced by Mesos (Table 10).

**Table 10:** Gamma test results for the OHF rootstock.

Output variables	Input combinations	Mask	Gamma (Γ)	Gradiant (A)	SE
Proliferation	*All inputs*	*111111*	0.5620	0.8669	1.5763
	*All inputs—KNO_3_*	*011111*	2.0077	0.4477	1.6985
	*All inputs—NH_4_NO_3_*	*101111*	2.6150	0.4354	0.8301
	*All inputs—Mesos*	*110111*	2.2833	0.5257	1.3597
	*All inputs—Micro*	*111011*	0.5965	1.2064	1.2197
	*All inputs—BA*	*111101*	**4.0178**	**0.1644**	**1.4234**
	*All inputs—IBA*	*111110*	2.0514	0.4577	1.1611
Shoot Length	*All inputs*	*111111*	0.2203	0.0957	0.0753
	*All inputs—KNO_3_*	*011111*	0.3648	0.0636	0.1170
	*All inputs—NH_4_NO_3_*	*101111*	0.3268	0.0765	0.1515
	*All inputs—Mesos*	*110111*	**0.5482**	**0.0405**	**0.1048**
	*All inputs—Micro*	*111011*	0.2010	0.1650	0.0535
	*All inputs—BA*	*111101*	0.3317	0.0952	0.1365
	*All inputs—IBA*	*111110*	0.3560	0.0707	0.1341
STN	*All inputs*	*111111*	48.9980	31.4820	42.7530
	*All inputs—KNO_3_*	*011111*	127.3200	127.3200	54.0010
	*All inputs—NH_4_NO_3_*	*101111*	116.5100	13.7040	42.9240
	*All inputs—Mesos*	*110111*	**184.0100**	**4.6396**	**51.9130**
	*All inputs—Micro*	*111011*	44.7660	46.7960	41.7740
	*All inputs—BA*	*111101*	106.2700	19.9260	58.8890
	*All inputs—IBA*	*111110*	130.9600	12.3230	51.3970
Vitrification	*All inputs*	*111111*	-17.7400	28.1300	25.9310
	*All inputs—KNO_3_*	*011111*	20.9180	19.3910	27.7170
	*All inputs—NH_4_NO_3_*	*101111*	29.4660	16.7610	45.3380
	*All inputs—Mesos*	*110111*	-0.0819	27.7110	31.5320
	*All inputs—Micro*	*111011*	**136.6100**	**-1.2718**	**35.4910**
	*All inputs—BA*	*111101*	-1.9649	28.8680	31.3780
	*All inputs—IBA*	*111110*	21.9860	16.8340	54.7800
Quality	*All inputs*	*111111*	0.0728	0.2768	0.2233
	*All inputs—KNO_3_*	*011111*	0.5746	0.1471	0.2216
	*All inputs—NH_4_NO_3_*	*101111*	0.6161	0.1341	0.1803
	*All inputs—Mesos*	*110111*	**1.1146**	**0.0408**	**0.3014**
	*All inputs—Micro*	*111011*	0.5159	0.2432	0.2885
	*All inputs—BA*	*111101*	0.4175	0.2268	0.2853
	*All inputs—IBA*	*111110*	0.8418	0.0829	0.3527

Note: Different combinations compared to study the input effects (inclusion and exclusion indicated by 1 or 0 in the mask).

GT data for Pyrodwarf rootstock are presented in Table 11. BA (Γ = 10.2920), micros (Γ = 0.7874), NH_4_NO_3_ (Γ = 166.410), KNO_3_ (Γ = 168.4400), and mesos (Γ = 1.4860) had the highest gamma values for PR, SL, STN, Vitri and QI. For this rootstock, each measured parameter was affected differently by the input factors.

**Table 11:** Gamma test results for Pyrodwarf rootstock.

Output Variable	Input combinations	Mask	Gamma (Γ)	Gradiant (A)	SE
Proliferation	*All inputs*	*111111*	2.1129	1.7403	4.0116
	*All inputs—KNO_3_*	*011111*	4.7818	0.9393	4.2339
	*All inputs—NH_4_NO_3_*	*101111*	6.8771	0.7586	1.6950
	*All inputs—Mesos*	*110111*	4.6236	1.2593	3.5021
	*All inputs—Micro*	*111011*	2.0421	2.5185	3.3655
	*All inputs—BA*	*111101*	**10.2920**	**0.0106**	**3.6452**
	*All inputs—IBA*	*111110*	5.2341	0.8714	2.9160
Shoot Length	*All inputs*	*111111*	-0.3573	0.3796	0.3801
	*All inputs—KNO_3_*	*011111*	0.4399	0.1785	0.3984
	*All inputs—NH_4_NO_3_*	*101111*	0.5431	0.1506	0.5246
	*All inputs—Mesos*	*110111*	0.2888	0.2331	0.4847
	*All inputs—Micro*	*111011*	**0.7874**	**0.2007**	**0.2793**
	*All inputs—BA*	*111101*	0.0427	0.3332	0.3510
	*All inputs—IBA*	*111110*	0.4607	0.1428	0.6549
STN	*All inputs*	*111111*	128.000	7.1974	52.0610
	*All inputs—KNO_3_*	*011111*	144.460	2.3894	31.0210
	*All inputs—NH_4_NO_3_*	*101111*	**166.410**	**-3.3839**	**48.6330**
	*All inputs—Mesos*	*110111*	141.900	3.5943	61.0130
	*All inputs—Micro*	*111011*	91.671	22.0500	42.6210
	*All inputs—BA*	*111101*	139.000	3.9727	57.7520
	*All inputs—IBA*	*111110*	150.2200	1.7223	49.7580
Vitrification	*All inputs*	*111111*	152.1200	1.1255	62.0530
	*All inputs—KNO_3_*	*011111*	**168.4400**	**-0.0008**	**79.8210**
	*All inputs—NH_4_NO_3_*	*101111*	147.4000	1.7224	19.3820
	*All inputs—Mesos*	*110111*	160.7700	0.7812	64.4780
	*All inputs—Micro*	*111011*	63.6200	38.3510	47.553
	*All inputs—BA*	*111101*	104.8200	18.6670	58.6660
	*All inputs—IBA*	*111110*	164.0400	1.0555	74.8700
Quality	*All inputs*	*111111*	1.3022	0.0347	0.4882
	*All inputs—KNO_3_*	*011111*	1.4856	0.0058	0.5191
	*All inputs—NH_4_NO_3_*	*101111*	1.3936	0.0003	0.1725
	*All inputs—Mesos*	*110111*	**1.4860**	**-0.0074**	**0.5018**
	*All inputs—Micro*	*111011*	0.6692	0.2912	0.2363
	*All inputs—BA*	*111101*	1.0645	0.1255	0.4323
	*All inputs—IBA*	*111110*	1.4855	0.0049	0.5013

## Discussion

In vitro culture medium composition directly influences explant growth. Recently, artificial intelligence has become a strong tool for analysis of plant tissue culture data and can accurately predict optimal media composition [10, 11, 18, 49]. Predictive models to examine the effect of macronutrients and hormonal content on the G × N15 *Prunus* explant growth and rooting were constructed successfully previously using an ANN modeling procedure [11, 18, 49]. In our earlier work on pear rootstocks (OHF 69 and Pyrodwarf), we used ANN-GA to forecast the optimum macronutrient concentrations for an in vitro medium and compared this method with stepwise regression modeling [10]. ANN-GA appeared promising for this purpose [10]. While ANN provides a good substitute for statistical regression, it does not identify any mathematical relationships between the input and output variables. Furthermore, the ANN technique requires a time consuming trial and error process in order to find network parameters such as hidden layers and number of neurons. Using other approaches, such as GP or decision tree algorithms, can potentially overcome some of the weaknesses of the ANN method. Use of GP as a GA has the advantage of generating prediction equations without presuming the previous form of the current relationships. While the decision tree methods are also characterized as tree structures, they generally explore a data space rather than a program space, as genetic algorithms do. GP methods can produce highly nonlinear models [73, 74] in comparison to linear M5’ tree models [75]. To the authors’ knowledge, decision tree algorithms have never been used to study the performance of in vitro plantlets. In this study, the M5’ algorithm [76] for developing the model tree, was used to model the effect of plant tissue culture media components on explant growth parameters. The M5’ model is a powerful soft computing technique which provides comprehensive mathematical equations that give users more insight into the parameters affecting the modeling process outcome [77, 78].

Previously we compared two algorithms, i.e. RBFNN and GEP, to test improved modeling techniques for predicting impacts of plant tissue culture media components on parameters of explant growth [40]. GEP was the more powerful and precise predicting method in addition to being practical.

In this study, we applied the M5’ tree model algorithm, one of the algorithms of the model tree (MT) technique, to predict optimized concentrations of plant tissue culture media components. The performance of the developed models was then compared with the performance of the GEP constructed models on the same data (Table 7). As mentioned above, MTs have been found previously to be more precise predicting methods than regression-based approaches and more clear than ANN methods [77]. The MT method has several advantages over other soft computing approaches such as ANN. Most importantly, MT does not need considerable trial and error inputs to attain the best model. However, the MT method has the key limitation that it produces only linear relationships. In addition, for more complex states, the transformation of input data may not be that simple and may not necessarily result in a several simple linear formulations [77]. According to the results, the optimized GEP approach provides better-fit calculation than M5’ tree model algorithm. In addition, it has been shown that the most effective optimization approach for optimizing GEP models was MOPSO.

One of the most important benefits of the GEP method, compared to other techniques, is the lack of need to assume the preceding form of the relationship in order to produce prediction equations. GP and its derivations have been used previously to determine complicated relationships fitting various experimental data [39, 79, 80]. For this method, better individuals are selected from among a population by using genetic variations and fitness functions. The genetic variations are introduced by genetic operators. GEP is a learning machine which is supposed to learn the relationship between variables in data groups. The difference between GEP and its precursors, GP and GA, is in their individual programming, which is as fixed length linear strings (chromosomes), shown eventually by expression trees in GEP. Whereas, in GP and GA, the expression of individuals is as fixed length linear strings (chromosomes) and nonlinear entities of different shapes (parse trees) and sizes, respectively. One of the strengths of GEP over GP and GA is that its genetic operators work at the chromosome level, which makes the formation of genetic diversity very simple. Another important point, that allows more complex programs with several sub-programs to be evolved, is the unique, multi-genic nature of GEP. GEP combines the benefits of both GP and GA while also overcoming some of their limitations [62]. During the past years, GEP has been used widely due to its high effectiveness and efficiency and the applications of GEP are very extensive and are rapidly increasing [81]. GEP algorithm, as one of the most efficient function mining algorithms, has been extensively used in classification, prediction, pattern recognition, and other research areas. GEP can mine an optimal function to deal with subsequent complicated tasks [82]. GEP is selected over ANNs model, since ANN is a black‐box model, while GEP can explain the developed prediction models with mathematical statements. The developed GEP models have been used to predict spring streamflow up to 5 months in advance with high accuracy [83]. GEP has been applied to specify the water quality and stress on rivers or lakes due to the pollutants found in the wastewater [84]. Because of the measurement conditions, data may have some missing values. Such problem can easily be solved by using GEP [84]. Results according to real data set show that the multiple gene expression programming and fuzzy expert system technique outperforms advanced acute hypotensive episode (a common serious postoperative complication in ICU, which may raise multiple system failure especially of cardiac and respiratory kinds, and even cause death) detection methods by obtaining high prediction accuracy [81].

Here, Explant growth in reaction to macronutrient concentration differed, based on pear rootstock genotype, as also occurred in our preceding study using ANN-based modeling which found the critical nutrients for optimal growth were NO_3_^−^ and NH_4_^+^ [10]. We therefore proposed use of ANN-based modeling to identify concentrations of macronutrients that would maximize PR and SL while minimizing STN and Vitri [10].

Detecting the optimum levels of minerals and hormones for a particular plant genotype is problematic due to their complicated interactions [85]. Moreover, the occurrence of physiological disorders such as hyperhydricity and necrosis during the proliferation stage of *Pyrus* genotypes has led researchers to use a variety of media for optimal growth. Developing an optimized culture medium likely will require use of a reliable mathematical modeling and optimization method [10, 11, 18, 49]. Various statistical procedures have been used previously to develop effective plant tissue culture media [10, 11, 18, 20, 49, 86].

The response surface method (RSM) and Multiple Linear Regression (MLR) have been used previously to develop optimal *in vitro* media for pear genotypes [10, 87]. Significantly higher accuracy of prediction was reported for ANN-GA models than for RSM and MLR [10, 88]. RBFNN-based models, along with optimization algorithms have also been effective.

The MNOGA optimized models obtained in this work indicate the importance of each nutrient or hormone for explant growth (Table 8). Previous studies on G × N15 *Prunus* rootstock using ANN-GA modeling identified the greater importance of NH_4_^+^, NO_3_^-^, PO_4_^2-^, Ca^2+^, and K^+^, relative to SO_4_^2-^, Mg^2+^, and Cl^−^, for in vitro proliferation [11]. MNOGA optimization of GEP models showed that high proliferation rate may result in reduced plantlet quality (Table 8). Performing multi-objective optimization techniques using GA and PSO on GEP models of Pyrodwarf showed that reducing BA and increasing NH_4_NO_3_, will increase QI and SL while decreasing the STN, Vitri, and PR (Table 9).

The role of the ratio of NO_3_^-^ to NH_4_^+^ in *Pyrus* rootstock micropropagation has been extensively discussed in the literature [89–92]. These results are in accordance with many preceding studies on various plant species [89, 90, 93, 94]. Macronutrient content of culture media is a major determining factor for explant growth of many plants, as was also found previously for pear rootstocks [10]. In this work we found a very small increase in NH_4_NO_3_ increased PR and Vitri of OHF explants while decreasing SL and QI (Table 9).

For Pyrodwarf, higher predicted PR obtained using MNOGA towards MOGA optimization method is a good point for selecting MNOGA as more appropriate model optimization method as STN and Vitri occurrences probability are also almost the same. Oppositely in OHF, higher predicted PR and also SL were found by using MOGA. Considering the lower QI and higher Vitri using MOGA may lead us to select MNOGA as more appropriate method for OHF, as well.

We used MOPSO as another multi-objective and efficient optimization method to achieve a more precise multi-objective optimization method for optimizing our models. Comparing the MOPSO results with MNOGA in Pyrodwarf revealed nearly the same PR, SL and QI using MNOGA and MOPSO, but STN and Vitri showed higher results using MOPSO (Tables 8 and 9). So, there is more probability of STN and Vitri occurrence using MOPSO optimized models in Pyrodwarf. The same comparison in OHF showed higher PR, SL and QI using MOPSO than MNOGA and lower STN was found using MOPSO towards MNOGA, but higher Vitri found using MOPSO to MNOGA (Tables 8 and 9). Accordingly, achieving higher proliferation rate can cover the low probability of Vitri occurrence (2.14). Consequently, in view of both results in Pyrodwarf and OHF, MOPSO can be considered as a more powerful GEP model multi-objective optimization method.

## Conclusion

The aim of our recent research [10, 11, 18, 49] has been to identify a more precise method for predicting optimum components of proliferation or rooting media. To the best of our knowledge, this is the first time that multi-objective optimization techniques have been used to optimize culture media composition. Here, GA and PSO optimized GEP models were applied to find optimum composition of pear rootstock media. RBFNN and GEP modeling showed that, consistent with former studies [89, 95, 96], decreasing the nitrogen content of the medium improves shoot growth and quality. We also found that lower nitrogen content combined with higher BA concentration results in higher PR, STN, Vitri and lower QI. Comparison of these results with our previous work [10, 11, 18, 49] indicates that use of multi-objective optimization methods leads to more accurate prediction.

Comparison of the M5’ model tree and GEP modeling algorithms showed that the GEP technique is more effective than M5’ model tree for assessing the interaction of culture medium factors on growth parameters. MOPSO optimization of the GEP model is introduced as an effective multi-objective optimization tool to achieve comprehensive results.

In general, GEP models are easy to use and provide unambiguous mathematical equations in forecasting accurately optimized culture media.

We suggest comparison of more multi-objective evolutionary algorithms like GSA for optimization of parameters to be studied in further researches.

## Supporting information

S1 Table Box–Behnken design of OHF micropropagation experiments and average values of the parameters used to characterize it. (DOCX)

S2 Table Box–Behnken design of Pyrodwarf micropropagation experiments and average values of the parameters used to characterize it. (DOCX)
