# Supplementary material for: High throughput mathematical modeling and multi-objective evolutionary algorithms for plant tissue culture media formulation: Case study of pear rootstocks
Source: PLoS One. 2020 Dec 18;15(12):e0243940. doi: 10.1371/journal.pone.0243940 (PMC7748151; doi:10.1371/journal.pone.0243940)
Supplement: S1 Table — (DOCX) [file pone.0243940.s001.docx]

| S1 Table. Box–Behnken design of OHF micropropagation experiments and average values of the parameters used to characterize it. | | | | | | | | | | | |
| --- | --- | --- | --- | --- | --- | --- | --- | --- | --- | --- | --- |
| Culture medium | Factor 1 | Factor 2 | Factor 3 | Factor 4 | Factor 5  (mgl^-1^) | Factor 6  (mgl^-1^) | PR | SL (cm) | STN | Vitri | QL |
|  | KNO_3_ | NH_4_NO_3_ | Mesos | Minors | BAP | IBA |  | | | | |
| 1 | 0 | -1 | 0 | 0 | -1 | 1 | 2.00 | 4.51 | 0 | 0 | 5.00 |
| 2 | 1 | 1 | 0 | -1 | 0 | 0 | 6.75 | 2.52 | 14.80 | 33.33 | 2.00 |
| 3 | 0 | -1 | 1 | 0 | -1 | 0 | 1.00 | 4.96 | 0 | 0 | 5.00 |
| 4 | 1 | 0 | -1 | 0 | 0 | -1 | 8.50 | 2.22 | 44.10 | 8.82 | 1.50 |
| 5 | 1 | 0 | 1 | 0 | 0 | -1 | 5.50 | 3.74 | 18.18 | 4.55 | 3.75 |
| 6 | 1 | 0 | 0 | -1 | 1 | 0 | 4.50 | 2.25 | 11.11 | 38.85 | 1.75 |
| 7 | 0 | -1 | 0 | 0 | 1 | -1 | 4.75 | 3.34 | 0 | 5.26 | 4.75 |
| 8 | 0 | 0 | -1 | 1 | 0 | -1 | 7.75 | 3.00 | 38.70 | 16.13 | 1.50 |
| 9 | 1 | 0 | 1 | 0 | 0 | 1 | 5.75 | 4.07 | 21.73 | 4.35 | 3.50 |
| 10 | 0 | 1 | 0 | 0 | 1 | 1 | 4.25 | 2.91 | 11.76 | 5.88 | 3.75 |
| 11 | 0 | 0 | 1 | 1 | 0 | 1 | 5.50 | 5.57 | 13.62 | 13.64 | 3.50 |
| 12 | -1 | -1 | 0 | 1 | 0 | 0 | 6.25 | 4.80 | 8.00 | 8.00 | 4.25 |
| 13 | 1 | -1 | 0 | 1 | 0 | 0 | 7.25 | 3.71 | 10.32 | 13.79 | 3.75 |
| 14 | 0 | 1 | -1 | 0 | 1 | 0 | 5.25 | 1.69 | 47.61 | 9.52 | 1.25 |
| 15 | -1 | 0 | -1 | 0 | 0 | -1 | 7.25 | 2.82 | 41.37 | 0 | 1.75 |
| 16 | 1 | -1 | 0 | -1 | 0 | 0 | 7.25 | 3.20 | 6.88 | 31.03 | 2.25 |
| 17 | 0 | -1 | 0 | 0 | 1 | 1 | 4.75 | 3.49 | 0 | 5.26 | 4.75 |
| 18 | 0 | 0 | 1 | -1 | 0 | 1 | 5.50 | 4.04 | 13.62 | 27.27 | 2.50 |
| 19 | 0 | -1 | 0 | 0 | -1 | -1 | 2.00 | 3.89 | 0 | 0 | 5.00 |
| 20 | 0 | 1 | 1 | 0 | 1 | 0 | 3.00 | 3.00 | 24.99 | 8.33 | 3.25 |
| 21 | 0 | -1 | -1 | 0 | -1 | 0 | 2.75 | 2.68 | 36.36 | 0 | 2.00 |
| 22 | 1 | 0 | -1 | 0 | 0 | 1 | 8.75 | 2.37 | 45.71 | 8.57 | 1.25 |
| 23 | 0 | 1 | -1 | 0 | -1 | 0 | 2.25 | 2.15 | 44.44 | 0 | 1.75 |
| 24 | 0 | -1 | -1 | 0 | 1 | 0 | 6.00 | 2.00 | 29.16 | 4.17 | 3.00 |
| 25 | 0 | 1 | 0 | 0 | 1 | -1 | 4.25 | 2.60 | 11.76 | 5.88 | 3.75 |
| 26 | -1 | -1 | 0 | -1 | 0 | 0 | 6.25 | 3.28 | 4.00 | 24.00 | 3.25 |
| 27 | -1 | 0 | 0 | 1 | -1 | 0 | 1.25 | 4.17 | 20.00 | 20.00 | 2.75 |
| 28 | -1 | 0 | 1 | 0 | 0 | 1 | 5.25 | 4.50 | 19.04 | 0 | 4.00 |
| 29 | 1 | 0 | 0 | 1 | 1 | 0 | 4.50 | 2.65 | 11.10 | 22.20 | 2.75 |
| 30 | 0 | 0 | -1 | -1 | 0 | 1 | 7.75 | 2.50 | 41.86 | 32.25 | 1.00 |
| 31 | -1 | 1 | 0 | -1 | 0 | 0 | 6.00 | 2.71 | 8.33 | 25.00 | 3.25 |
| 32 | 0 | 0 | 1 | -1 | 0 | -1 | 5.50 | 3.90 | 13.62 | 31.82 | 2.00 |
| 33 | -1 | 0 | 0 | -1 | 1 | 0 | 4.00 | 2.02 | 6.25 | 31.25 | 2.50 |
| 34 | 0 | 0 | 1 | 1 | 0 | -1 | 5.25 | 5.00 | 14.28 | 13.83 | 3.50 |
| 35 | 1 | 0 | 0 | 1 | -1 | 0 | 1.75 | 3.07 | 14.28 | 14.29 | 3.50 |
| 36 | 0 | 0 | -1 | -1 | 0 | -1 | 7.75 | 2.20 | 38.64 | 29.03 | 1.75 |
| 37 | 1 | 0 | 0 | -1 | -1 | 0 | 1.75 | 2.31 | 14.28 | 42.86 | 1.25 |
| 38 | 0 | 1 | 0 | 0 | -1 | 1 | 1.50 | 3.88 | 16.66 | 0 | 3.75 |
| 39 | 0 | 1 | 0 | 0 | -1 | -1 | 1.50 | 3.36 | 16.66 | 0 | 3.75 |
| 40 | -1 | 0 | 1 | 0 | 0 | -1 | 5.25 | 4.27 | 19.04 | 0 | 3.50 |
| 41 | 1 | 1 | 0 | 1 | 0 | 0 | 6.75 | 2.75 | 18.50 | 18.52 | 3.25 |
| 42 | 0 | -1 | 1 | 0 | 1 | 0 | 3.75 | 3.50 | 13.32 | 0 | 3.75 |
| 43 | 0 | 0 | -1 | 1 | 0 | 1 | 8.00 | 3.39 | 43.75 | 12.50 | 1.25 |
| 44 | -1 | 0 | 0 | 1 | 1 | 0 | 4.00 | 2.90 | 12.50 | 18.75 | 3.50 |
| 45 | 0 | 1 | 1 | 0 | -1 | 0 | 1.00 | 4.01 | 25.00 | 0 | 3.00 |
| 46 | -1 | 0 | -1 | 0 | 0 | 1 | 7.50 | 3.33 | 39.99 | 0 | 2.50 |
| 47 | -1 | 1 | 0 | 1 | 0 | 0 | 6.00 | 4.29 | 12.48 | 8.33 | 3.75 |
| 48 | -1 | 0 | 0 | -1 | -1 | 0 | 1.25 | 3.04 | 0 | 20.00 | 3.00 |
| MS | 1 | 1 | 1 | 1 | 2.5 | 0.1 | 3.25 | 3.08 | 7.69 | 0 | 4.25 |
| WPM | 1 | 1 | 1 | 1 | 2.5 | 0.1 | 2.25 | 4.25 | 11.11 | 22.22 | 3.00 |
| QL | 1 | 1 | 1 | 1 | 2.5 | 0.1 | 3.00 | 2.81 | 0 | 0 | 5.00 |
