# Supplementary material for: High throughput mathematical modeling and multi-objective evolutionary algorithms for plant tissue culture media formulation: Case study of pear rootstocks
Source: PLoS One. 2020 Dec 18;15(12):e0243940. doi: 10.1371/journal.pone.0243940 (PMC7748151; doi:10.1371/journal.pone.0243940)
Supplement: S2 Table — (DOCX) [file pone.0243940.s002.docx]

| S2 Table. Box–Behnken design of Pyrodwarf micropropagation experiments and average values of the parameters used to characterize it. | | | | | | | | | | | |
| --- | --- | --- | --- | --- | --- | --- | --- | --- | --- | --- | --- |
| Culture medium | Factor 1 | Factor 2 | Factor 3 | Factor 4 | Factor 5  (mgl^-1^) | Factor 6  (mgl^-1^) | PR | SL (cm) | STN | Vitri | QL |
|  | KNO_3_ | NH_4_NO_3_ | Mesos | Minors | BAP | IBA |  | | | | |
| 1 | 0 | -1 | 0 | 0 | -1 | 1 | 2.43 | 5.32 | 0 | 5.33 | 4.68 |
| 2 | 1 | 1 | 0 | -1 | 0 | 0 | 7.45 | 1.23 | 40.17 | 49.64 | 1.00 |
| 3 | 0 | -1 | 1 | 0 | -1 | 0 | 1.78 | 4.93 | 0 | 6.43 | 4.45 |
| 4 | 1 | 0 | -1 | 0 | 0 | -1 | 8.98 | 2.46 | 37.32 | 21.97 | 1.75 |
| 5 | 1 | 0 | 1 | 0 | 0 | -1 | 8.18 | 2.51 | 36.09 | 21.40 | 1.78 |
| 6 | 1 | 0 | 0 | -1 | 1 | 0 | 5.13 | 0.96 | 31.68 | 30.25 | 1.50 |
| 7 | 0 | -1 | 0 | 0 | 1 | -1 | 6.33 | 3.77 | 0 | 5.10 | 4.53 |
| 8 | 0 | 0 | -1 | 1 | 0 | -1 | 10.18 | 0.81 | 17.44 | 23.35 | 2.80 |
| 9 | 1 | 0 | 1 | 0 | 0 | 1 | 8.50 | 2.75 | 37.14 | 21.48 | 1.85 |
| 10 | 0 | 1 | 0 | 0 | 1 | 1 | 5.55 | 3.47 | 22.51 | 37.84 | 1.65 |
| 11 | 0 | 0 | 1 | 1 | 0 | 1 | 9.33 | 1.58 | 16.09 | 23.04 | 2.80 |
| 12 | -1 | -1 | 0 | 1 | 0 | 0 | 10.73 | 1.51 | 10.01 | 7.43 | 3.88 |
| 13 | 1 | -1 | 0 | 1 | 0 | 0 | 9.03 | 1.77 | 20.20 | 9.19 | 3.48 |
| 14 | 0 | 1 | -1 | 0 | 1 | 0 | 6.00 | 3.15 | 24.95 | 39.17 | 1.45 |
| 15 | -1 | 0 | -1 | 0 | 0 | -1 | 11.73 | 2.25 | 10.28 | 17.69 | 3.58 |
| 16 | 1 | -1 | 0 | -1 | 0 | 0 | 9.03 | 2.08 | 19.11 | 12.96 | 3.30 |
| 17 | 0 | -1 | 0 | 0 | 1 | 1 | 6.35 | 4.25 | 0 | 5.88 | 4.43 |
| 18 | 0 | 0 | 1 | -1 | 0 | 1 | 9.18 | 1.85 | 14.36 | 28.47 | 2.58 |
| 19 | 0 | -1 | 0 | 0 | -1 | -1 | 2.23 | 4.81 | 0 | 4.86 | 4.60 |
| 20 | 0 | 1 | 1 | 0 | 1 | 0 | 5.10 | 3.15 | 24.02 | 38.25 | 2.68 |
| 21 | 0 | -1 | -1 | 0 | -1 | 0 | 3.08 | 4.63 | 3.99 | 6.76 | 4.23 |
| 22 | 1 | 0 | -1 | 0 | 0 | 1 | 9.15 | 2.85 | 38.00 | 22.15 | 1.68 |
| 23 | 0 | 1 | -1 | 0 | -1 | 0 | 2.10 | 3.07 | 25.16 | 38.29 | 1.53 |
| 24 | 0 | -1 | -1 | 0 | 1 | 0 | 7.03 | 3.54 | 5.04 | 7.05 | 4.13 |
| 25 | 0 | 1 | 0 | 0 | 1 | -1 | 5.38 | 3.10 | 20.51 | 38.11 | 1.70 |
| 26 | -1 | -1 | 0 | -1 | 0 | 0 | 10.55 | 1.83 | 9.27 | 10.18 | 3.83 |
| 27 | -1 | 0 | 0 | 1 | -1 | 0 | 1.80 | 1.34 | 8.45 | 19.23 | 3.35 |
| 28 | -1 | 0 | 1 | 0 | 0 | 1 | 9.80 | 2.44 | 10.46 | 17.34 | 3.45 |
| 29 | 1 | 0 | 0 | 1 | 1 | 0 | 4.80 | 0.91 | 34.91 | 23.90 | 1.75 |
| 30 | 0 | 0 | -1 | -1 | 0 | 1 | 10.08 | 1.36 | 17.41 | 29.10 | 2.28 |
| 31 | -1 | 1 | 0 | -1 | 0 | 0 | 9.40 | 0.99 | 13.11 | 45.71 | 1.45 |
| 32 | 0 | 0 | 1 | -1 | 0 | -1 | 9.13 | 1.53 | 14.78 | 28.22 | 2.53 |
| 33 | -1 | 0 | 0 | -1 | 1 | 0 | 5.75 | 1.25 | 7.40 | 25.21 | 3.13 |
| 34 | 0 | 0 | 1 | 1 | 0 | -1 | 9.23 | 1.32 | 14.09 | 22.74 | 2.70 |
| 35 | 1 | 0 | 0 | 1 | -1 | 0 | 1.15 | 1.32 | 35.00 | 23.00 | 1.65 |
| 36 | 0 | 0 | -1 | -1 | 0 | -1 | 9.95 | 1.12 | 16.58 | 28.65 | 2.38 |
| 37 | 1 | 0 | 0 | -1 | -1 | 0 | 1.00 | 1.55 | 32.50 | 30.00 | 1.78 |
| 38 | 0 | 1 | 0 | 0 | -1 | 1 | 1.20 | 3.14 | 23.00 | 36.50 | 1.70 |
| 39 | 0 | 1 | 0 | 0 | -1 | -1 | 1.03 | 2.95 | 19.50 | 36.50 | 1.80 |
| 40 | -1 | 0 | 1 | 0 | 0 | -1 | 9.75 | 2.04 | 9.23 | 17.16 | 3.50 |
| 41 | 1 | 1 | 0 | 1 | 0 | 0 | 7.78 | 1.04 | 41.00 | 43.23 | 1.00 |
| 42 | 0 | -1 | 1 | 0 | 1 | 0 | 5.60 | 3.84 | 0 | 6.72 | 4.45 |
| 43 | 0 | 0 | -1 | 1 | 0 | 1 | 10.38 | 1.06 | 19.04 | 23.13 | 2.65 |
| 44 | -1 | 0 | 0 | 1 | 1 | 0 | 5.83 | 0.84 | 8.60 | 19.72 | 3.28 |
| 45 | 0 | 1 | 1 | 0 | -1 | 0 | 1.00 | 3.23 | 25.00 | 37.50 | 1.63 |
| 46 | -1 | 0 | -1 | 0 | 0 | 1 | 12.08 | 2.25 | 12.00 | 17.80 | 3.48 |
| 47 | -1 | 1 | 0 | 1 | 0 | 0 | 9.50 | 0.76 | 15.56 | 40.25 | 1.63 |
| 48 | -1 | 0 | 0 | -1 | -1 | 0 | 1.63 | 1.64 | 7.20 | 26.07 | 2.95 |
| MS | 1 | 1 | 1 | 1 | 2.5 | 0.1 | 3.53 | 3.99 | 14.23 | 10.23 | 3.60 |
| WPM | 1 | 1 | 1 | 1 | 2.5 | 0.1 | 2.28 | 2.57 | 6.47 | 3.25 | 4.18 |
| QL | 1 | 1 | 1 | 1 | 2.5 | 0.1 | 2.95 | 2.62 | 2.73 | 0 | 4.78 |
